# Combatting rumors around the French election: the memorability and effectiveness of fact-checking articles

**DOI:** 10.1186/s41235-023-00500-2

**Published:** 2023-07-13

**Authors:** Lisa K. Fazio, Min Kyung Hong, Raunak M. Pillai

**Affiliations:** Department of Psychology and Human Development, Vanderbilt University, 230 Appleton Place #552, Jesup 105, Nashville, TN 37203 USA

**Keywords:** Misinformation, Correction, Fact check, Debunk

## Abstract

Across four studies, we examined the how design decisions influenced the effectiveness of fact-checking articles created by CrossCheck France during the 2017 French election. We measured both memory for the article and belief in the false rumor. We saw no difference in fact check efficacy based on the type of headline (question vs negation) or the number of newsroom logos present around the article (one, four, or seven). In addition, informative design features such as an icon identifying the type of misinformation were ignored by readers. Participants failed to remember many of the details from the article, but retrieval practice was beneficial in reducing forgetting over a 1-week delay. In both US and French samples, reading the fact check decreased belief in the false information, even 1 week later. However, the articles were much more effective in the US sample, who lacked relevant prior knowledge and political beliefs. Overall, fact-checking articles can be effective at reducing belief in false information, but readers tend to forget the details and ignore peripheral information.

## Introduction

The spread of and belief in misinformation is a major problem in today’s society. While misinformation has always been present, today’s interconnected world means that it spreads farther and faster than ever before (Vosoughi et al., 2018). One product that has emerged to fight against false information is the fact-checking article. First developed by organizations such as FactCheck.org and PolitiFact, these articles lay out the evidence for why a claim is false. In this way, they are similar to the refutation texts that are used to combat science misconceptions (e.g., Sinatra & Broughton, 2011; Tippett, 2010) or historical misunderstandings (Donovan et al., 2018) in educational settings. Often written by journalists, fact checks use basic principles of journalism (e.g., reliance on primary sources) to check the truthfulness of politicians’ statements and other widely circulated claims (see Graves, 2016 for a history of the format).

Over the past 8 years, there has been an explosion of these fact-checking organizations. While only 44 fact-checking projects existed in 2014, the Duke Reporters’ Lab documented 391 projects across 105 countries in 2021 (Stencel et al., 2022). In the USA, these fact checks are published by major newspaper organizations such as the Associated Press and The Washington Post, and play a vital role in Facebook’s attempts to curb the spread of misinformation on their platform (Lyons, 2018). This increased prevalence means that readers often now encounter fact-checking articles even when they were not explicitly searching for them. However, it is still unclear how people interpret and remember these articles.

One concern has been around backfire effects and whether presenting a fact check can actually make readers double-down and believe the false information more. More accurately described as the worldview backfire effect, the idea is that if an article challenges a deeply held belief, readers are motivated to defend their existing worldview and this internal defense may increase their belief in the false information (see Lewandowsky et al., 2012 for a review). Recent evidence suggests that these backfire effects are actually extremely rare and possibly nonexistent (Swire-Thompson et al., 2020). Many of the effects have failed to replicate (Haglin, 2017; Wood & Porter, 2019) and if the worldview backfire effect does occur, it does so in very limited circumstances.

Instead, corrections are generally effective in reducing belief in false information (see Chan et al., 2017; Walter & Murphy, 2018 for meta-analyses). However, the specific type of correction varies tremendously across studies from simple labels of true/false (e.g., Ecker et al., 2020; Swire et al., 2017a, 2017b) to a few sentences or a paragraph (e.g., Aird et al., 2018; Swire et al., 2017a, b). Only occasionally are participants given full articles (e.g., Nyhan et al., 2019) and when they are, the articles are rarely presented as they would be viewed in real-life.

Yet, fact-checking organizations have put a lot of thought into how they present information to the reader and the design features they use. In the current studies, we experimentally tested the effectiveness of two visual features which were designed by the CrossCheck France organization to improve the effectiveness of the articles: (1) including the logos of the multiple newsrooms which had contributed to the fact check and (2) phrasing the article’s headline as a question rather than a statement. In addition, we examined the effect of reading the fact-checking article on belief in the false information and how well the article was remembered by the readers both immediately and after a 1-week delay. The research was conducted in collaboration with CrossCheck in order to inform their future fact-checking efforts and to increase our knowledge of how fact-checks are read, processed and remembered.

Across four studies, we examined readers’ reactions to actual fact-checking articles published by CrossCheck France around the 2017 French presidential election. CrossCheck was a nonprofit collaborative journalism project that allowed French news organizations to jointly research and publish fact-checking articles during the 10 weeks preceding the election. Over 40 newsrooms participated and articles were published both on the CrossCheck website and in the news organizations’ own newspapers and websites.

In order to visually signal that the articles were collaborative and non-partisan, each organization who signed off on a fact check added their newsroom’s logo to the side of the article. This innovative design feature was intended to increase readers’ trust in the article. There are two interrelated reasons why these newsroom logos may make the articles more effective. First, the logos may signal a level of expert consensus around the information contained in the fact check. In other domains, information about the level of expert consensus around information on topics such as vaccinations or climate change (e.g., that 97% of scientists believe climate change is caused by human action) can help in correcting misconceptions (Lewandowsky et al., 2013; van der Linden et al., 2015). Similarly, by signaling widespread agreement around the fact check among various news outlets, the newsrooms logos may help increase belief change in the political information examined here. Second, the logos may make the source of the information seem more credible. Even if readers don’t particularly trust a single newsroom, they may trust one of the other newsrooms signing off on the fact check. This practice may be particularly important in France, where the news landscape is especially fragmented (less than 20% of people name the same organization as their top source for news) and where there are large political divides in which outlets are trusted (Pew Research Center, 2019a). Overall trust in news media is also low in France, with only 4% of French adults saying they have a lot of trust in the news media (Pew Research Center, 2019a). Accordingly, the logos may increase the likelihood that the information is associated with a source the reader trusts. This source credibility is an important feature of persuasive messaging (Pornpitakpan, 2004) and can ultimately affect the acceptance of corrective information (Berinsky, 2017; Guillory & Geraci, 2013). In sum, if the addition of newsroom logos increases the credibility of the fact check or the perception of expert consensus around the fact check, articles with more logos should be more effective in reducing belief in misinformation. We test this hypothesis directly in Experiments 3 and 4.

A second innovative feature of the CrossCheck articles was that they always presented headlines in the form of a question. That is, instead of titling the fact check “Macron does not want to get rid of family allowances,” the article would be titled “Does Macron want to get rid of family allowances?” The question format was designed to both increase the readers’ curiosity (which can increase learning; Gruber & Ranganath, 2019) and reduce the number of times the false rumor was repeated in the context of a statement. We know that repetition increases the perceived truth of statements (Fazio et al., 2015; Unkelbach et al., 2019), but it is still unclear how that repetition interacts with attempts to correct information. Some researchers suggest that repeating a false claim within a correction can increase belief (Schwarz et al., 2007), perhaps because people remember that the false claim was stated in an earlier sentence, but not that it was in the context of being negated. In fact, recent studies suggest that the effects of repetition on belief are smaller with questions than statements (Mattavelli et al., 2023) implying that the question format may prevent negative effects of repeating the false information. However, others have found that repeating the false information is neutral or even beneficial for correction (Ecker et al., 2017; Walter & Tukachinsky, 2020). In Experiments 1 and 2, we manipulated the headline format to examine if the question format was more effective.

The present studies were designed to answer three main questions: (1) Does headline style matter for fact check memory or effectiveness? (2) Do additional newsroom logos increase a fact check’s effectiveness or readers’ trust in the article? and (3) How well do readers remember the fact-checking articles? In addition, in order to examine the effect of prior knowledge and beliefs, we tested the fact checks both in the USA (Experiments 1 and 2), where readers had very little knowledge of the French election, and in France, where readers were both more knowledgeable and more invested in the topics (Experiments 3 & 4). To preview, we found that the fact checks were effective in both countries (more so in the US), but that readers were unaffected by visual design features such as the type of headline and the number of newsroom logos. In addition, readers did not remember many of the design features but could recognize many of the specific details from the article, even after a 1-week delay.

## Experiment 1

### Method

#### Participants

Two hundred and seventeen adults (*M*_age_ = 36.8 years; age range = 19–76) in the USA were recruited online using Amazon’s Mechanical Turk. Using CloudResearch (Litman et al., 2017), we restricted the sample to participants in the USA and blocked duplicate IP addresses. One hundred and ten participants were randomly assigned to the headline-negation condition, and 107 participants were assigned to the headline-question condition.

#### Design

The current study employed a between-subjects design with two headline conditions and 10 possible fact-checking articles. Participants were randomly presented with an article from a set of 10 articles, and headlines were randomly assigned to be phrased as a question (headline-question condition) or as a negation statement (headline-negation condition). In addition, for the accuracy ratings, time of rating was manipulated within-subject (pretest, posttest).

### Materials

#### Fact-checking articles

Ten articles were selected from CrossCheck France (https://web.archive.org/web/20181204201007/https://crosscheck.firstdraftnews.org/france-en/), a website designed to counter misinformation surrounding the 2017 French presidential election and its candidates. Articles addressed prevalent rumors related to the presidential campaign that had spread widely on social media. The contents of each report were reviewed or “cross-checked” by multiple news organizations. Logos of the newsrooms who had participated in this verification process were displayed beside the article. There were also visual icons, such as *True*, *False*, *Caution*, *Insufficient Evidence* and *Attention*, denoting the credibility of the rumor discussed in the article (with most rumors labeled as either False or Attention). *False* rumors were completely false, while *Attention* was used for claims that were partially true but exaggerated or presented in a misleading fashion (e.g., an actual photo from 2014 used as evidence for an event in 2017). If an article was marked as *False*, an additional label was added that categorized the nature of misinformation as either *manipulated*, *manufactured*, *misattributed*, *misleading*, *misreported*, or *satire* (see Wardle, 2017 for definitions).

**Fig. 1:**
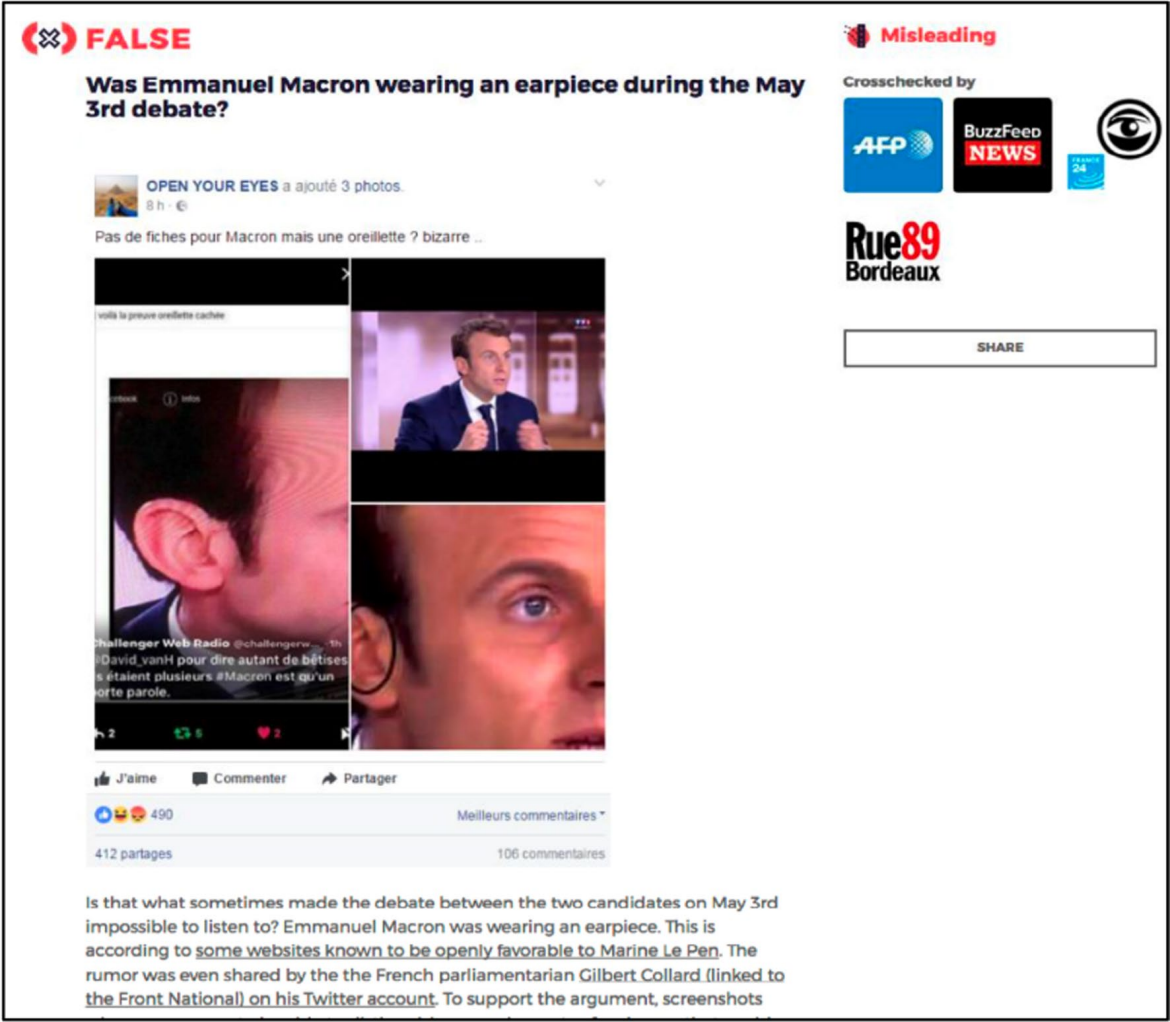
Screenshot of the top of one of the articles used in Experiment 1

We chose ten articles as stimuli using the following selection criteria: the headline ended with a question mark, the article contained around 250 words, and the article displayed four to eight newsroom logos (for a complete list of selected articles, see https://osf.io/n27pt/). Five of the articles dealt with rumors about Emmanuel Macron, while others focused on rumors about other candidates (one each for Ferrand, Fillon, and Le Pen) or French society as a whole (two articles). See Fig. 1 for a sample article.

The headline of each article posed its main topic, a rumor, in a question form (e.g., “Was Emmanuel Macron wearing an earpiece during the May 3^rd^ debate?”). For each article, we created a second version of its headline in which the rumor was negated in the form of a statement (e.g., “Emmanuel Macron was not wearing an earpiece during the May 3rd debate.”). Therefore, the title of each article was presented either as a question or as a negation of the rumor discussed in the article. As mentioned above, each article contained three visual features. First, each article in the study was marked as *False* or *Attention*, representing the truth value of rumor discussed in the article. Out of the 10 articles, eight were labeled as False and two Attention. Second, False articles displayed an additional label indicating the type of misinformation. For example, if the content of the misinformation was baseless or entirely fabricated, it was labeled as “manufactured”. Of the eight False articles, three were Misleading, two were Manufactured, two were Manipulated (e.g., involved photoshopped imagery), and one was Satire. Lastly, the right side of the screen displayed logos of each media outlet that endorsed the reporting underlying the fact-checking article. The selected articles each included 4—8 logos (*M* = 5.7, *SD* = 1.63).

#### Pre- and posttest questionnaire

Prior to reading a fact-checking article, participants completed a pretest questionnaire. They were given a set of 10 statements corresponding to the misinformation addressed by the 10 articles. For each statement, participants were asked to rate its accuracy on an 11-point Likert scale. The scale ranged from 0 = very inaccurate to 10 = very accurate. The statements were created by modifying the articles’ headlines (e.g., “Was Emmanuel Macron wearing an earpiece during the May 3^rd^ debate?”) into a restatement of the rumor addressed (e.g., “Emmanuel Macron was wearing an earpiece during the May 3^rd^ debate”). After reading a fact-checking article, participants were again presented with the corresponding rumor and asked to rate its accuracy (posttest questionnaire). While participants rated all 10 rumors on the pretest, they only rated the rumor corresponding to the article they read on the posttest.

#### Memory questionnaire

We assessed participants’ memory for the article with a questionnaire containing eight questions pertaining to different aspects of the article (for a complete list of questions, see https://osf.io/n27pt/). Each question was presented on a separate screen, and participants were not allowed to revisit their previous answers. The presentation order of the questions was identical for all participants (see Table 1). First, participants indicated if the authors labeled the rumor as True, False, or Attention. They were then asked about two visual details from the article: the label at the top-right corner of the article, indicating the type of misinformation it addressed (e.g., Misleading), and the number of newsroom logos beside the article. Next, participants’ memory for the article was assessed with an open-ended, free recall question. Finally, participants answered four multiple-choice questions about the rumor and the specific evidence that was used to support or refute it. These last four questions differed depending on the article presented, but always included three answer choices. Sample questions are presented in Table 1.

**Table 1:**
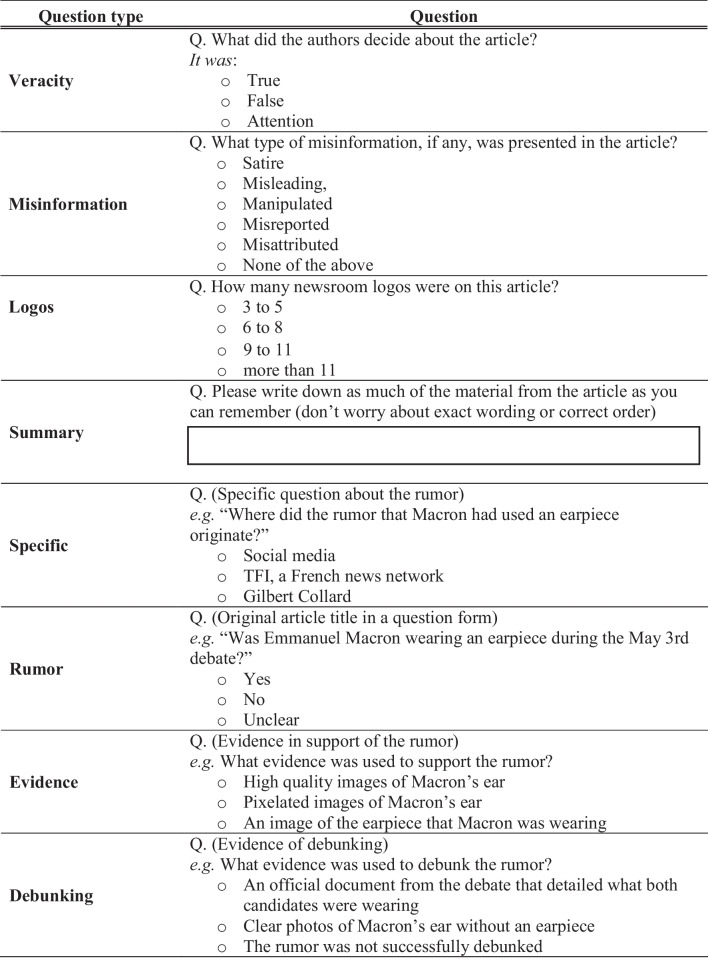
Sample questions from one version of the memory questionnaire

Question types Veracity, Misinformation, Logos, and Summary were identical across all articles, while Specific, Rumor, Evidence and Debunking changed across articles

#### Procedure

Participants started with the pretest questionnaire and rated the accuracy of all 10 rumors (pre-rating). Next, they were told that they were about to see an article that fact-checks a rumor spread during the recent French election. They were asked to read the article as they would a typical article on the internet and were told that they would answer questions about the article afterward. Immediately after reading the article, participants were presented with the rumor from that article and were asked to rate its accuracy (post-rating). Then, participants completed the memory questionnaire described above. The entire experiment was self-paced, so participants could spend as much time as they wanted viewing the article and answering the questions.

### Results

#### Accuracy ratings

We first examined participants’ accuracy ratings of the rumor both before and after reading the fact-checking article. While participants provided accuracy ratings for all 10 rumors before reading the fact-check, we focus here on only their ratings of the rumor relevant to their assigned article. Because it was unclear what accuracy rating would be correct for the articles labeled “Attention”, we examined only the eight rumors which were clearly false. This left 176 participants in the analysis (86 in the headline-question and 90 in the headline-negation condition). As a reminder, the accuracy scale ranged from 0 = very inaccurate to 10 = very accurate. Thus, we predicted that reading the fact-checking article should decrease participants’ ratings. According to a simulation-based sensitivity analysis conducted using simR (Green & MacLeod, 2016), our final sample of 176 participants provides 80% power, 95% CI [77%, 82%] to detect an interaction between headline type and time of at least 1.5 in the mixed effects model reported below. The simR package uses Monte Carlo simulation to compute the statistical power that a given model has to detect a given effect of a user-specified size. In our sensitivity analyses, we used our existing model/sample size, and varied the effect size until we identified the smallest effect size that we had 80% power to detect.

As shown in Table 2, participants were more likely to believe that the relevant misinformation was false after reading the fact-checking article. However, there was no effect of reading the headline as a question versus a statement. These patterns were confirmed statistically using linear mixed effects models. We used the “buildmer” package (Voeten, 2020) to find the maximal model that would still converge (as suggested by Barr et al. (2013)) and then tested the model using the “lme4” package (Bates et al., 2015) and the “lmerTest” package (Kuznetsova et al., 2017) to provide p-values using Satterthwaite’s method in R (R Core Team, 2020). We follow this process for each of the mixed effects models presented in this manuscript.

**Table 2:** Average accuracy ratings split by time of rating and headline type (Experiment 1)

Headline	Pretest	Posttest
Question	4.02 (2.69)	1.97 (2.76)
Negation	4.34 (2.90)	1.69 (2.65)
*M*	4.19 (2.79)	1.82 (2.70)

The accuracy scale ranged from 0 = very inaccurate to 10 = very accurate. Standard deviations are in parentheses

The final model predicted accuracy ratings as a function of the fixed effects of time (pretest, posttest), headline type (question, negation) and their interaction, along with random intercepts for participant and article and by-article random slopes for time. Both time and headline type were contrast coded and centered at zero (pretest = − 0.5, posttest = 0.5; question = − 0.5, negation = 0.5). As shown in Table 3, accuracy ratings were significantly higher on the pretest than posttest, but there was no significant effect of headline format or their interaction.

**Table 3:** Mixed-effects model testing the effect of test time and headline type on accuracy ratings (Experiment 1)

Fixed effects	Estimate	*SE*	*df*	*t* Value	*p* Value
Intercept	**3.004**	**0.199**	**7.211**	**15.065**	** < 0.001**
Time [pretest = − 0.5, posttest = 0.5]	− **2.366**	**0.521**	**6.96**	**4.538**	**0.003**
Headline [question = − 0.5, negation = 0.5]	0.028	0.320	171.082	0.087	0.931
Time*Headline	− 0.604	0.491	168.532	1.229	0.221

Random effects	Variance	SD
*Participant* (intercept)	1.860	1.364
*Article* (intercept)	0.114	0.338
Time	1.697	1.303

Model was fit to 352 accuracy ratings from 176 participants across 8 articles. Bolded values indicate significant effects

#### Memory questions

For both the multiple-choice questions and the free recall summary, we analyzed the full sample of 217 participants. For ease of analysis, we split the multiple-choice memory questions into three categories: main idea, visual features, and specific details. The main idea category contained two questions about whether the rumor was true or false (Veracity and Rumor in Table 1). Visual features examined whether participants could remember the type of misinformation and the number of newsroom logos beside the article (Misinformation and Logos). Specific details examined participants memory for the details of the article (Specific, Evidence, Debunk).

As shown in Table 4, the headline format (question or negation) did not affect participants’ memory for the fact-checking article. To test the pattern statistically, we conducted three separate linear mixed effects models predicting memory accuracy on each question type based on the fixed effect of headline type and random intercepts for each article.^1^ Participants in both conditions did not differ in their ability to remember the main idea, *b* = 0.01, *t*(206.58) = 0.30, *p* = 0.763, the visual features, *b* = 0.001, *t*(206.8) = 0.30, *p* = 0.976, or specific details, *b* < 0.001, *t*(211.1) = 0.018, *p* = 0.986, of the presented article.

**Table 4:** Proportion of correct responses on the memory questions split by headline format (Experiment 1)

Memory question	Question	Negation
Veracity	0.63 (0.49)	0.70 (0.46)
Rumor	0.69 (0.46)	0.66 (0.48)
*M* main idea	0.66 (0.40)	0.68 (0.42)
Misinformation	0.30 (0.46)	0.35 (0.48)
Logos	0.58 (0.50)	0.57 (0.50)
*M* visual features	0.44 (0.36)	0.46(0.38)
Specific	0.64 (0.48)	0.70 (0.46)
Evidence	0.67 (0.47)	0.63 (0.49)
Debunk	0.69 (0.46)	0.67 (0.47)
*M* specific details	0.67 (0.31)	0.67 (0.27)

Standard deviations are in parentheses. Chance performance would be 0.33 for all questions except for misinformation (0.17) and logos (0.25). Models were fit to 217 observations across 217 participants and 10 articles

#### Free recall summary

Next, we examined what participants recalled in response to the summary question (free recall). Participants’ responses were coded based on the number of “idea units” recalled (similar to the coding scheme used by Roediger & Karpicke, 2006). Each article was determined to have 10–18 idea units (*M* = 12.6). These idea units were often full sentences from the article (e.g., “But, the images are so highly magnified and pixelated that they are near impossible to decipher.”), but sentences that contained multiple key ideas were separated into multiple idea units (e.g., “However, in very clear photos taken during the same debate” and “no earpiece appears in the each in question of the En Marche! Candidate.”). The idea units were then used as a scoring rubric for two independent coders who examined each of the participants’ responses. For each response, the raters marked whether each idea unit was present (1) or absent (0).

The participants’ exact wording did not matter, but their response had to accurately capture the entire relevant idea unit to be counted as correct. For example, in order to get credit for the following idea unit, “This accusation is according to some websites known to be openly favorable to Marie Le Pen,” a response must include the main idea unique to this segment (i.e., acknowledging that the rumor came from a biased source) regardless of the exact wording. Therefore, a response such as “the rumor came from a source biased toward Marie Le Pen” or “the rumor originated from a website that is partial to his opponent” would be counted as “present”: both responses reflect that a participant correctly recalled the context in which the main idea “websites known to be openly favorable to Marie Le Pen” was mentioned in the article. On the other hand, a response that contains word fragments from the idea unit but fails to deliver the main idea such as “the rumor came from some websites,” would be marked as absent. It fails to acknowledge that the rumor came from a source known to be biased against Macron. As a result, each participant received a series of 1’s or 0’s for every idea unit from the article they read. To assess interrater reliability, Cohen’s kappa (Cohen, 1960) was calculated for each article. On average across all ten articles, the two coders showed a “substantial agreement” (Landis & Koch, 1977), *kappa* = 0.709. A third coder (the second author) then resolved any discrepancies between the two coders. Using these final scores, we calculated the proportion of idea units recalled by each participant.

Overall, participants recalled very few of the idea units from the fact-checking articles, but there was no significant difference in the proportion of idea units recalled between the headline-question (*M* = 0.17, *SD* = 0.15) and headline-negation (*M* = 0.16, *SD* = 0.15) conditions, *b* = − 0.01, *t*(207.68) = 0.55, *p* = 0.585. (Again, tested using a linear mixed effects model with headline type as a fixed effect and random intercepts for each article).

#### Influences on accuracy ratings

Finally, we examined whether participants’ memory for the article was related to their accuracy scores. We again restricted the analysis to the eight false rumors and conducted a linear regression with posttest accuracy as the dependent measure and pretest accuracy rating, accuracy on the specific detail questions, and proportion of idea units recalled in the summary as predictors. Due to a large right skew, we performed a natural log transformation on the idea unit measure (first adding 0.01 to all values to eliminate zeros). We again examined this question using a linear mixed effects model. As shown in Table 5, participants who recalled more information during the open-ended summary and answered more specific detail questions correctly gave lower accuracy ratings to false claims on the posttest.

**Table 5:** Mixed-effects model examining influences on delayed posttest accuracy ratings (Experiment 1)

Fixed effects	Estimate	*SE*	*df*	*t* Value	*p* Value
Intercept	2.471	0.743	35.29	3.326	0.002
Specific details	− **3.180**	**0.614**	**169.6**	− **5.178**	** < 0.001**
Summary (ln)	− **0.458**	**0.177**	**8.895**	− **2.590**	**0.029**
Pretest rating	0.100	0.065	162.3	1.529	0.128

Random effects	Variance	*SD*	*r*
*Article* (intercept)	0.855	0.925	
Summary (ln)	0.110	0.331	0.84

Model was fit to 176 accuracy ratings from 176 participants across 8 articles. Bolded values indicate significant effects. Correlation is between the term in the given row and the preceding random-effects term

### Discussion

Overall, we found no evidence that the headline format (statement vs question) affected readers belief in the false claim or their memory for the article. However, the fact-checking articles were very effective in reducing belief in the false rumors. Participants’ accuracy ratings on average decreased from above 4 on the pretest to below 2 on the posttest (on a 0–10 scale). Note that participants’ initial ratings of the false rumors were already relatively low, thus the articles are not correcting strongly believed claims. They are, however, increasing the accuracy of readers’ beliefs. While readers remembered very little of the article when asked to freely recall as much as they could (~ 16% of idea units), they were well above chance at recognizing specific details on a multiple-choice test^2^ [67% correct vs. chance of 33%, *t*(216) = 17.00, *p* < 0.001]. In addition, we found preliminary evidence for a relation between memory for the details of the article and its effectiveness. The evidence is purely correlational and thus could exist for many reasons, but its presence suggests that increasing memory for the fact-checking article may increase its corrective power.

These results lead to the two goals of Experiment 2. First, we wanted to examine the durability of the correction. Would readers remember that the rumor was false 1 week later? Second, we were interested in whether improving memory for the specific details of the article would increase its effectiveness. Retrieval practice or testing is a very effective way of increasing learning (see Karpicke, 2012 for a review), thus we examined the effect of the immediate memory questionnaire on participants’ accuracy ratings 1 week later. We predicted that retrieval practice would enhance memory for details of the article, and this memory would help participants remember why the key claim is false. As a result, participants who took an immediate memory test should have lower accuracy ratings 1 week later. As in Experiment 1, participants completed the pretest ratings, read the article, and then completed the posttest rating. Half of the participants then completed the immediate memory questionnaire. One week later, participants were asked to complete a second survey where they gave a delayed posttest accuracy rating and completed the memory questionnaire. We also added some additional demographic questions and asked participants how often they had read news about the French election.

## Experiment 2

### Method

#### Participants

Four hundred and seventy-seven adults in the USA were recruited online using Amazon’s Mechanical Turk (*M*_age_ = 36 years; age range = 19–74 years). Using CloudResearch (Litman et al., 2017), we restricted the sample to participants in the USA and blocked duplicate IP addresses. Of the 477 participants who participated in Session 1, 341 participants also completed Session 2 and are included in the analyses below. The majority of the participants reported having never read about the French presidential election (46%), followed by having read about the election one or two times (27%), once a week (15%), multiple times a week (7%), and once a month (5%).

#### Design

The current study employed a 2 (headline: question, negation) × 2 (immediate memory test: yes, no) × 10 (fact-checking article) between-subjects design and was conducted over two sessions. Accuracy ratings were measured three times for each participant (pretest, immediate posttest, delayed posttest).

#### Materials

The materials used in this experiment were identical to those in Experiment 1.

### Procedure

**Fig. 2:**
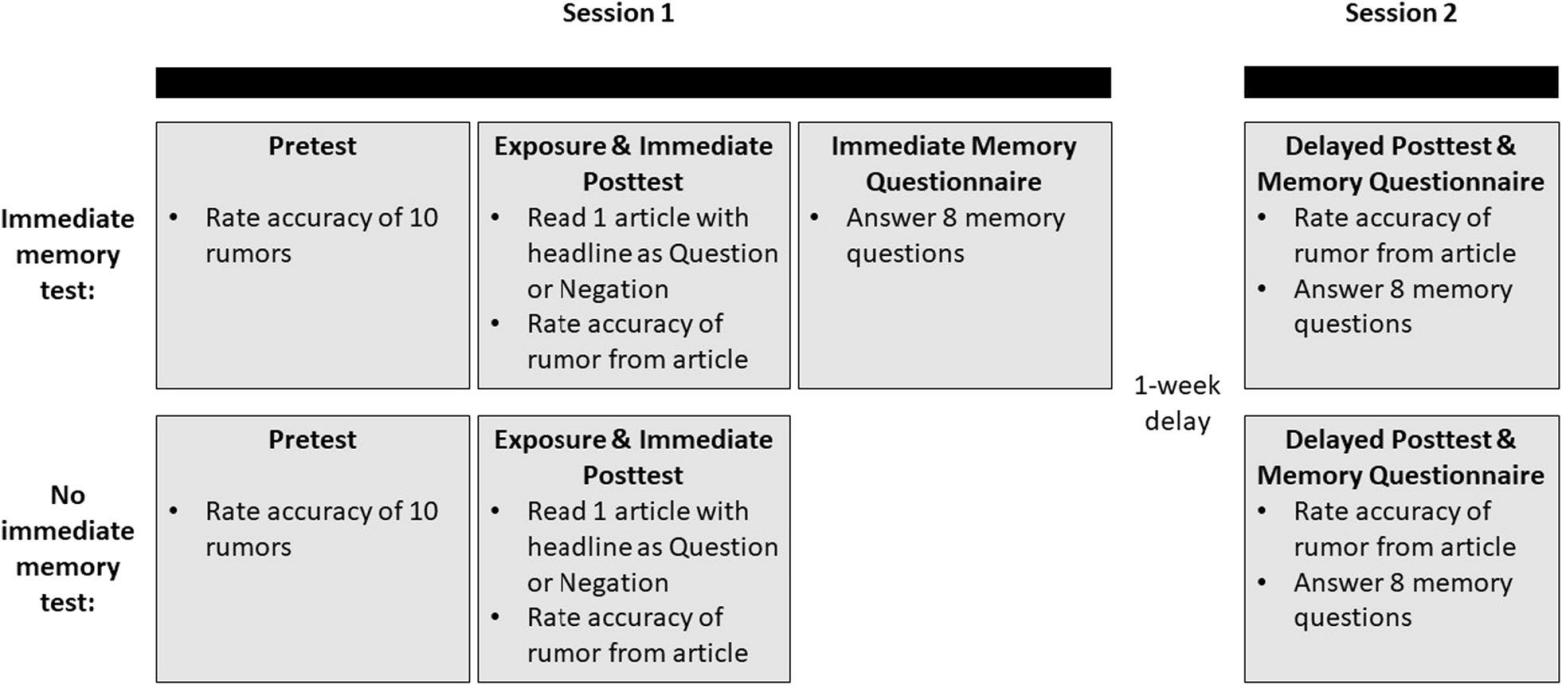
Study design for Experiment 2

Participants were tested in two sessions, separated by 1 week. See Fig. 2 for an illustration of the design. The procedure for the first session was nearly identical to Experiment 1, except that only half of the participants received the immediate memory questionnaire. Participants were first presented with 10 rumor statements in a random order and were asked to rate the accuracy of each statement. Then, they read a randomly chosen fact-checking article and completed the posttest rating. Participants in the no immediate test condition ended Session 1 there and did not answer the memory questionnaire, whereas participants in the immediate test condition completed the memory questionnaire as in Experiment 1.

Participants who completed Session 1 were contacted via email 1 week later with a link to the second survey. The instructions for the survey began by briefly reminding participants of the article they read a week ago (e.g., “Please answer the following questions about the article you read last week about Emmanuel Macron’s earpiece during a debate”). After reading the instructions, participants were asked to rate the accuracy of the rumor (delay-rating) and complete the memory questionnaire. Participants were then asked to indicate “How often did you read news about the French election while it was occurring?” with the answer choices of multiple times a week, once a week, once a month, only once or twice, and never. The survey ended with an optional demographics survey that asked about their age, gender, ethnicity, and education level.

### Results

Our analyses focus on only the 341 participants who completed both sessions.

#### Accuracy ratings

For the accuracy ratings, we again restricted our analyses to the eight false articles, leaving 276 participants in the analysis (137 who took both an immediate and delayed test and 139 who took only the delayed test). This sample provides 81% power, 95% CI [78%, 83%] to detect an interaction between headline type and time on the immediate test of at least 1.33 (simulation-based power analysis using simr). Consistent with Experiment 1, mixed-effects regression models revealed that the type of headline did not affect accuracy ratings on the immediate (question *M* = 1.87, negation *M* = 1.70; *b* = − 0.22, *t*(7.10) = − 0.39, *p* = 0.710) or delayed posttest (question *M* = 2.76, negation *M* = 2.45, *b* = − 0.30, *t*(272.15) = − 0.866, *p* = 0.387). Thus, we collapsed over headline type in the analyses below. (Full results are available at https://osf.io/n27pt/.) Table 6 presents mean accuracy ratings on the pretest, immediate posttest and delayed posttest.

**Table 6:** Mean accuracy ratings for the false rumors split by timepoint and immediate test condition (Experiment 2)

Immediate memory test	Pretest	Immediate posttest	Delayed posttest
No	3.98 (2.66)	1.98 (2.94)	2.82 (2.96)
Yes	4.84 (2.88)	1.58 (2.66)	2.39 (2.90)
*M*	4.41 (2.80)	1.78 (2.80)	2.60 (2.93)

The accuracy scale ranged from 0 = very inaccurate to 10 = very accurate. Standard deviations are in parenthesis

Overall, reading the fact check reduced participants’ belief in the rumor and that decrease persisted 1 week later. We confirmed these observations by fitting a linear mixed effects model predicting accuracy ratings as a function of the fixed effects of time (pretest, posttest, delayed test), testing (immediate test, no immediate test), and their interaction, along with random intercepts for participants and article. Time was dummy coded with pretest as the baseline and testing was contrast coded and centered at zero (no immediate test = − 0.5, immediate test = 0.5). As shown in Table 7, accuracy ratings significantly decreased from the pretest to the immediate posttest and remained below baseline at the delayed posttest.

**Table 7:** Mixed-effects model testing the effect of test time and test condition on accuracy ratings (Experiment 2)

Fixed effects	Estimate	*SE*	*df*	*t* value	*p* value
Intercept	**4.416**	**0.202**	**15.515**	**21.855**	** < 0.001**
Time2 [posttest vs. pretest]	− **2.630**	**0.198**	**548**	**13.291**	** < 0.001**
Time3 [delayed test vs. pretest]	− **1.807**	**0.198**	**548**	**9.133**	** < 0.001**
Testing [no test = − 0.5, immed test = 0.5]	**0.0864**	**0.340**	**673.344**	**2.544**	**0.011**
Time2*testing	− **1.259**	**0.396**	**548**	**3.182**	**0.002**
Time3*testing	− **1.293**	**0.396**	**548**	**3.267**	**0.001**

Random effects	Variance	SD
*Participant* (intercept)	2.545	1.595
*Article* (intercept)	0.095	0.309

Model was fit to 828 accuracy ratings from 276 participants across 8 articles. Bolded values indicate significant effects.

Unexpectedly, we observed a significant main effect of testing such that participants in the immediate testing group rated the rumors as slightly more accurate than participants who did not take an immediate test (*M*_*immed test*_ = 2.936 vs.* M*_*no test*_ = 2.927) In addition, this main effect of testing group was qualified by an interaction with both contrasts for rating timepoint (posttest vs. pretest and delayed test vs. pretest). To probe this interaction, we reran the model in Table 7 twice with the testing variable dummy coded (i.e., no test = 0, immediate test = 1 or vice versa), allowing us to interpret the time contrast terms as simple effects of time for the no test or the immediate test group. In these analyses we find that both effects of time were smaller in magnitude, though still significant, for the participants who received no test (*b*_posttest_ = − 2.00, *t*(548.00) = − 7.12, *p* < 0.001; *b*_delayed_ = − 1.16, *t*(548.00) = − 4.13, *p* < 0.001) relative to the participants who did receive a test (*b*_posttest_ = − 3.26, *t*(548.00) = − 11.69, *p* < 0.001; *b*_delayed_ = − 2.45, *t*(548.00) = − 8.80, *p* < 0.001). However, both the pretest and immediate posttest accuracy ratings occurred before participants were assigned to the testing or no testing condition. Thus, the difference is likely due to random chance or a preexisting difference between participants who did or did not receive an immediate memory test.

A priori, we had predicted that accuracy ratings would increase over time from the immediate to the delayed posttest, and that participants who took the immediate memory test would forget less over time. Thus, the increase in their accuracy ratings over the 1-week delay would be smaller than for participants in the no immediate test group. To assess this hypothesis, we reran the model in Table 7, setting the reference level for the time contrasts to the immediate posttest. While we observed a significant increase in ratings from the immediate to delayed posttest (*b* = 0.82, *t*(548.00) = 4.16, *p* < 0.001), this effect was not qualified by an interaction with testing condition as we had predicted (*b* = − 0.03, *t*(548.00) = − 0.09, *p* = 0.932).

#### Memory questions

We again split the multiple-choice memory questions into three categories: main idea, visual features, and specific details. For ease of analysis, we present only the mean proportion correct for each category in the main text, but the full results are presented at https://osf.io/n27pt/. As in Experiment 1, mixed-effects regression models for each of these three measures revealed no effect of headline format on memory accuracy on the immediate test (largest *t*(9.43) = 2.02, *p* = 0.073) and no effect after the 1-week delay (largest *t*(9.17) = 0.91, *p* = 0.388). Thus, we collapsed across headline format in the analyses below. The full results are presented at https://osf.io/n27pt/.

**Fig. 3:**
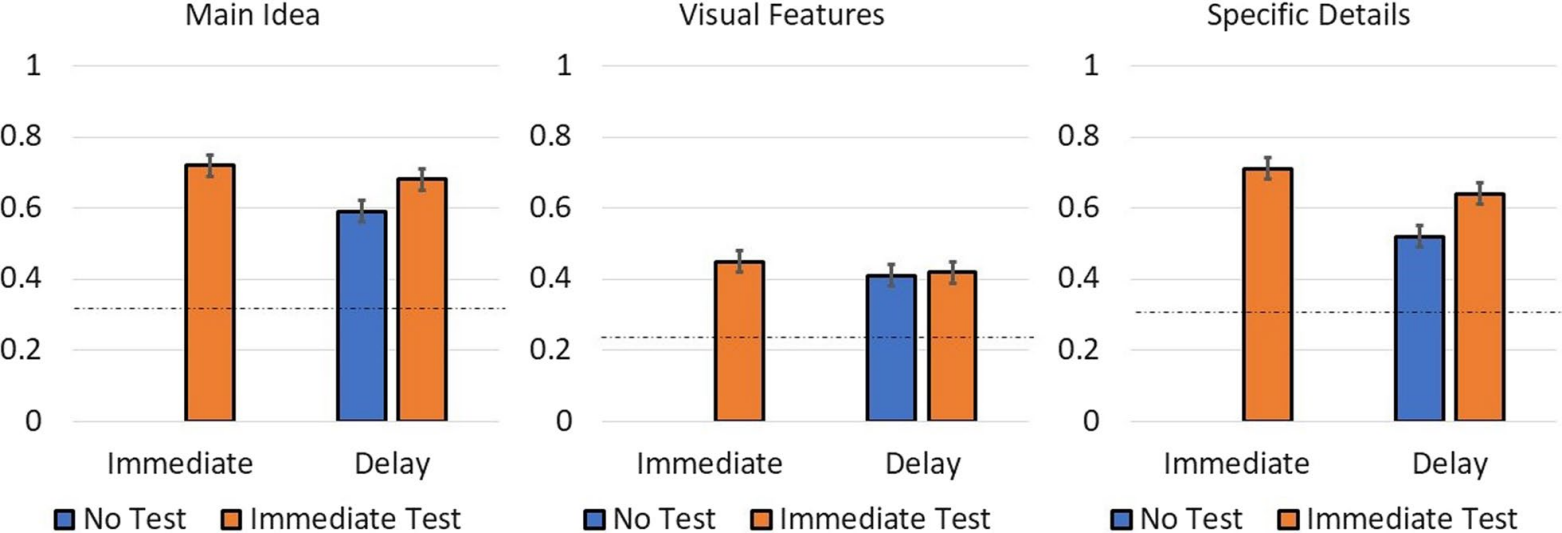
Proportion correct on the different types of memory question split by delay and whether there was an immediate test (Experiment 2). The dashed line indicates chance performance

Figure 3 presents the proportion correct for each category of question on both the immediate and delayed memory test. As predicted, participants forgot some information over the 1-week delay, but an initial test decreased forgetting over time.

##### Effect of delay

We first examined the effect of a 1-week delay on responses by comparing the immediate test accuracy for the immediate memory test group with the delayed test accuracy of the group without an immediate test. To do this, we used mixed-effects regression models for each measure with test time contrast-coded (immediate test = − 0.5, delayed test = 0.5). One week later, participants were less accurate at answering questions about the main idea of the article, (*b* = − 0.11,* t*(330.64) = − 2.79, *p* = 0.006), and the specific details, (*b* = − 0.18, *t*(9.879) = − 5.28, *p* < 0.001). There was no significant decrease in accuracy for the visual features, (*b* = − 0.06, *t*(9.01) = − 1.46, *p* = 0.177).

##### Effect of testing

Next, we examined how taking an immediate test affected accuracy on the delayed test. Here we again used mixed-effects regressions to compare the performance of the two testing groups (no test = − 0.5, immediate test = 0.5) on the delayed test. The immediate test group was more accurate on the specific details questions, (*b* = 0.11, *t*(331.35) = 3.53, *p* < 0.001), despite not receiving feedback on the immediate memory test. However, an initial memory test resulted in only a marginally significant increase in accuracy on the delayed test for the main idea (*b* = 0.07, *t*(330.71) = 1.85, *p* = 0.065) and had no effect for visual features, *t*(9.44) = 0.74, *p* = 0.480.

#### Free recall summary

We again had two independent raters code the number of idea units recalled in each summary and the 2^nd^ author resolved all discrepancies (*kappa* = 0.789 on the immediate test and 0.751 on the delayed test). A similar pattern emerged for the free recall summaries. A mixed-effects regression model showed that participants recalled more of the article’s contents immediately after reading (*M* = 0.19, *SD* = 0.15) as compared to 1-week later (*M* = 0.09, *SD* = 0.11; *b* = 0.10, *t*(9.34) = − 5.69, *p* < 0.001). In addition, participants who had received an earlier memory test recalled more of the article 1-week later (*M* = 0.12, *SD* = 0.12) than participants who did not take an immediate test (*M* = 0.09, *SD* = 0.11; *b* = 0.04, *t*(331.15) = 3.37, *p* < 0.001). Finally, as in Experiment 1, we did not find a significant effect of headline type on free recall summary scores (*b* = 0.01, *t*(332.37) = 1.14, *p* = 0.25).

#### Influences on delayed accuracy ratings

Finally, we examined predictors of participants’ delayed accuracy ratings. Among participants who took an immediate memory test, we examined whether participants’ immediate memory for the article was related to their delayed accuracy scores. In a second analysis, we examined how participants’ time spent reading about the French election affected their delayed accuracy ratings. To focus on how reading the fact-checking article changed beliefs, we controlled for pretest accuracy ratings in both analyses and again focused on only the eight false articles.

For our first analysis examining the influence of initial memory accuracy on later accuracy scores, there was no random effects structure that converged after using “buildmer.” Thus, we instead fit a linear regression using “lm” in R to predict accuracy ratings from the proportion of specific details questions answered correctly on the immediate test, the log-transformed number of idea units correctly recalled from the article, and pre-test accuracy ratings. As expected, pre-test scores predicted posttest scores (*b* = 0.19, *t*(135) = 2.40, *p* = 0.018). Critically, after controlling for pre-test scores, greater memory for the details of the article on the immediate test was related to lower accuracy ratings 1 week later, as measured by the proportion of accurate responses to specific detail multiple-choice questions (*b* = − 2.72, *t*(135) = − 3.09, *p* = 0.002) and the log-transformed number of correctly recalled idea units (*b* = − 0.460, *t*(135) = − 2.65, *p* = 0.009).

In addition, as shown in Table 8, participants who were more familiar with the French election were less affected by reading the fact-checking article. That is, after controlling for pretest rating, participants who reported more familiarity with the French election gave higher accuracy ratings to the false rumor 1 week later.

**Table 8:** Mixed-effects model testing the effect of election news familiarity on accuracy ratings, controlling for pre-test accuracy ratings (Experiment 2)

Fixed effects	Estimate	*SE*	*df*	*t* value	*p* value
Intercept	**0.999**	**0.467**	**66.33**	**2.138**	**0.036**
Pre-rating	**0.225**	**0.062**	**272.9**	**3.657**	** < 0.001**
Election news	**0.301**	**0.129**	**270.1**	**2.332**	**0.020**

Random effects	Variance	*SD*
*Article* (intercept)	0.292	0.541

Model was fit to 276 accuracy ratings from 276 participants across 8 articles. Bolded values indicate significant effects

### Discussion

Replicating Experiment 1, headline format did not alter participants’ accuracy ratings or memory for the article. Consistent with past evidence of the benefits of retrieval practice, participants who took the immediate memory questionnaire better remembered the article 1 week later. But, that experimental manipulation did not lead to lower accuracy ratings for the misinformation on the delayed posttest. There was a similarly small increase in accuracy ratings for the misinformation over the week delay for both the participants who did and did not take the immediate test. As in Experiment 1, participants with better memory also rated the false rumors as being less accurate, but experimentally increasing participants’ memory through retrieval practice was not beneficial. We return to this surprising result in the general discussion. More encouragingly, the overall benefits of reading the fact-checking article were still seen 1 week later. Participants rated the false rumors as less accurate after reading the article both immediately and 1 week later.

In Experiments 1 and 2, we purposely used participants from the USA who were, in general, unfamiliar with the details of the French election. This choice allowed us to examine the effectiveness of the articles and design features when participants had little prior knowledge and no pre-existing partisan beliefs. However, the next step was to add those complications by examining the effectiveness of these fact-checking articles among French participants. Following the results from Experiment 2 where the articles were less effective for participants with more electoral knowledge, we expected the fact checks to be less effective for these French participants.

In addition, Experiment 3 was designed to test the relation between the number of newsroom logos presented beside the fact-checking article and the article’s credibility and effectiveness. The CrossCheck project was designed to increase readers’ trust by indicating that multiple news organizations agreed about the contents of the article. However, it is not yet known whether readers are sensitive to this type of cue and if it will affect their beliefs. As such, we manipulated whether each fact check featured 1, 4 or 7 newsroom logos and explicitly asked participants about the credibility of the article.

## Experiment 3

### Methods

#### Participants

Six hundred and twenty-three CloudResearch (formerly TurkPrime) panel members (*M*_age_ = 36.7 years; age range = 18–76 years) completed the survey. All participants were members of the CloudResearch’s French panel and currently living in France. Two hundred and fourteen participants saw one newsroom logo, 203 participants saw four newsroom logos, and 206 participants saw seven newsroom logos along with the article. The experiment was conducted in the end of March 2018, approximately 11 months after the French election. In the second round of the French presidential election, 38% of the participants reported voting for Macron, 21% voted for Le Pen, 7% voted for a different candidate and 34% did not vote.

#### Design

We varied the number of newsroom logos in a between-subjects design. Participants were randomly presented with an article from a set of 10 fact-checking articles and were assigned to one of three possible conditions (number of logos: 1-logo, 4-logos, and 7-logos). The timing of the accuracy ratings was again manipulated within-subjects (pretest, immediate posttest).

#### Materials

The stimuli were mostly identical to those from Experiments 1 and 2 with a few changes. First, since the participants were residents of France, the entire experiment was presented in French. For the fact-checking articles, we used the French versions of each article which were posted on the Crosscheck website (https://web.archive.org/web/20181204213903/https://crosscheck.firstdraftnews.org/france-fr/). The ratings and memory questionnaires were first translated from English into French by a bilingual research assistant and then independently back-translated from French to English by a second translator who was a native French speaker fluent in English (translation/back-translation method following the procedure recommended by Brislin, 1980).

Second, each article was modified from its original version so that the number of logos presented with the article varied across conditions. For each article, we constructed three different versions: 1-logo, 4-logos, and 7-logos. The type and display sequence of logos were fixed such that participants in the 1-logo condition always saw *AFP*, the 4-logos condition saw *AFP, Le Monde, French 24* and *Libération* and those in 7-logo condition saw *AFP, Le Monde, French 24, Libération, Explicite, Rue89 Lyon,* and *LCI*. Therefore, participants were presented with one of 30 possible choices at random (10 news articles x three versions).

Finally, we made two changes to the memory questionnaire. First, we omitted the free recall summary question due to concerns about our ability to accurately score answers that would have been provided in French (given the lack of native French speakers on our research team). Second, we changed the visual features question about the number of logos presented with the article from a multiple-choice question to free response. Participants were asked “How many logos were on this article?” and could type in any number as a response. This change allowed us to more accurately measure participants’ memory for the logos rather than constraining their responses. The other six multiple-choice questions remained unchanged.

##### Opinion questionnaire

In addition to the rating and memory questionnaires, we assessed participants’ opinions on the article they read and their political beliefs (for the full questionnaire and translations, see https://osf.io/n27pt/). Each question was presented separately on its own screen and participants were not allowed to revisit their previous answers once they advanced to the next question.

First, participants were asked about their opinions on the debunking of rumor in the article. They were asked to rate the credibility of article (“In your view, how credible was the debunk you just read?”) on a 5-point scale where 1 = not credible at all, 2 = slightly credible, 3 = moderately credible, 4 = very credible, and 5 = extremely credible. Then, participants were asked if they believed that the article was biased (“Was the debunk you read biased?) to which they answered either “yes, biased” or “no, not biased”. Participants were then asked if the bias reflected a certain political belief (“If you answered yes to the previous questions, do you think the bias was right, left, or other?), to which they were given four answer choices: right, left, center, and another political belief. Then, participants were asked to rate the degree of bias (“Would you say the debunk was extremely biased, somewhat biased, or slightly biased?”) on a 5-point scale where 1 = not biased at all and 5 = extremely biased.

Next, participants were asked to describe their political beliefs (“Thinking about politics these days, how would you describe your political viewpoint? How would you describe your political beliefs”) by choosing from one of the following options: Very right, Right, A little right, Center, A little left, Left, Very left, and I don’t know. Political viewpoint in France is typically considered on a right/left scale rather than a conservative/liberal scale (Fleury & Lewis-Beck, 1993). Participants were then asked to indicate which candidate they voted for in the first round of French presidential election by choosing from one of the following options: Emmanuel Macron, Marie Le Pen, François Fillon, Jean-Luc Mélenchon, a different candidate, and I didn’t vote. Lastly, they were asked to indicate which candidate they voted for during the second round of the election. The choices were: Emanuel Macron, Marine Le Pen, a different candidate, and I didn’t vote.

#### Procedure

As in Experiment 1, participants started with the pretest accuracy ratings before reading one of the fact-checking articles, providing a posttest accuracy rating and completing the memory questionnaire. Finally, they completed the opinion questionnaire asking about the credibility of the article and their political affiliation.

### Results

#### Accuracy ratings

Focusing on the 8 false rumors (N = 500), reading the fact-checking article again reduced participants’ belief in the rumor (Table 9). However, the number of newsroom logos did not affect participants’ accuracy ratings. We again confirmed these observations with a linear mixed effects model predicting accuracy ratings as a function of the fixed effect of time (pretest = − 0.5, posttest = 0.5), logos (1 [reference level], 4, 7) and their interaction, along with random intercepts for participants and article and by-article random slopes for time. A simulation-based sensitivity power analysis indicated 81% power, 95% CI [78 = 9%, 84%] to detect an interaction between time and the 1 vs 7 logo comparison at least 0.95.

**Table 9:** Mean accuracy ratings, logo estimates, credibility and bias ratings for the false rumors split by number of logos presented (Experiment 3)

Number of newsroom logos	Pretest accuracy	Immediate posttest accuracy	Logo estimate	Credible	Bias
One	4.75 (3.34)	4.02 (3.41)	2.81 (1.94)	2.99 (1.15)	0.49 (0.50)
Four	4.86 (3.36)	4.10 (3.52)	3.01 (1.86)	3.14 (1.17)	0.49 (0.50)
Seven	4.47 (3.09)	3.79 (3.24)	3.60 (2.27)	2.98 (1.13)	0.54 (0.50)
*M*	4.69 (3.26)	3.97 (3.39)	3.14 (2.06)	3.03 (1.15)	0.50 (0.50)

The accuracy scale ranged from 0 = very inaccurate to 10 = very accurate. Credibility scale ranged from 1 = not at all credible to 5 = extremely credible. Bias is the proportion of participants indicating that the article was biased. Standard deviations are in parenthesis

As shown in Table 10, the mean accuracy rating decreased by 0.739 points from the pretest to the posttest. However, it is important to note that the decrease was much smaller within this French sample (standardized regression coefficient *β* = − 0.22) than in Experiments 1 & 2 (*β* = − 0.79 and *β* = − 1.81, respectively). In addition, the number of logos did not alter the effectiveness of the article; adding additional logos did not affect accuracy ratings relative to the article with one logo.

**Table 10:** Mixed-effects model testing the effect of number of logos and test time on accuracy ratings (Experiment 3)

Fixed effects	Estimate	*SE*	*df*	*t* value	*p* value
Intercept	**4.382**	**0.308**	**15.662**	**14.215**	** < 0.001**
Time [pretest = − 0.5, posttest = 0.5]	− **0.739**	**0.295**	**21.594**	**2.502**	**0.0204**
Logos4	0.109	0.314	490.429	0.347	0.729
Logos7	− 0.254	0.312	490.119	0.812	0.417
Time*Logos4	− 0.029	0.344	490.604	0.085	0.932
Time*Logos7	0.054	0.343	490.126	0.160	0.873

Random effects	Variance	*SD*
*Participant* (intercept)	5.751	2.398
*Article* (intercept)	0.378	0.614
Time	0.238	0.488

Model was fit to 1000 accuracy ratings from 500 participants across 8 articles. Bolded values indicate significant effects

#### Memory questions

The full results are presented at https://osf.io/n27pt/, but we focus here on the mean proportion correct for each category, collapsed across the number of newsroom logos. The French participants correctly answered 51% (*SD* = 41) of the Main Idea questions and 51% (*SD* = 31) of the Specific Details questions. Since one of the visual features questions was specifically about the number of logos presented next to the article, we examined that question separately. (Memory for the other visual detail, the misinformation-type label, was again poor, *M* = 25% correct, *SD* = 43). As shown in Table 9, while participants did slightly adjust their estimates based on the number logos they saw, they tended to always think there were ~ 3 logos presented. (Six participants skipped the logo question and are excluded from the analysis). A mixed effects regression model indicated a small, but significant, effect of the number of logos presented on participants’ logo estimates (*b* = 0.13, *t*(606.56) = 3.93, *p* < 0.001)*.*

#### Credibility and bias

We also examined if the number of newsroom logos affected readers’ perceptions of credibility and bias by conducting mixed-effects regression models predicting credibility and bias from the number of logos (1 [reference level], 4, 7). As shown in Table 9, the number of logos had no effect on readers’ perceptions of the article’s credibility, (*b*_4 vs. 1_ = 0.14, *t*(612.44) = 1.32, *p* = 0.189; *b*_7 vs. 1_ = − 0.02, *t*(611.58) = − 0.14, *p* = 0.888), or if they thought the article was biased, (*b*_4 vs. 1_ = 0.002, *t*(611.90) = 0.042, *p* = 0.987; *b*_7 vs. 1_ = 0.05, *t*(611.20) = 1.10, *p* = 0.272).

#### Influences on accuracy ratings

Finally, we examined how participants’ political affiliation, their memory for the specific details of the article, and their perceptions of the article’s credibility affected their posttest accuracy ratings. All analyses were conducted using separate mixed-effects regression models that included a term to control for participants’ ratings of the item at pretest.

**Fig. 4:**
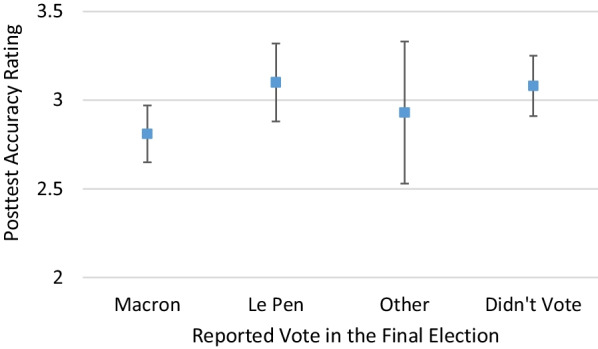
Marginal means of posttest accuracy ratings split by reported vote, controlling for pretest accuracy ratings (Experiment 3). Error bars are 95% confidence intervals. The accuracy scale ranges from 1 = very inaccurate to 10 = very accurate

We examined two different measures of political affiliation. The first was their political viewpoint (right to left) and the second was who they voted for in the second round of the presidential election. For the viewpoint analysis, we excluded 168 participants who answered “I don’t know” and treated the scale as continuous with 1 = very right and 7 = very left. Participants who were more left-leaning gave lower accuracy ratings on the posttest (*b*** = **− 0.30, *t*(364.56) = − 3.51, *p* < 0.001). Looking at participants’ voting records, we found a significant effect of candidate support such that Le Pen voters gave higher (less correct) accuracy ratings than Macron voters (*b* = 0.90, *t*(494.34) = 2.61, *p* = 0.009), as shown in Fig. 4 and Table 11.

**Table 11:** Mixed-effects model testing the effects of candidate choice on posttest accuracy ratings (Experiment 3)

Fixed effects	Estimate	*SE*	*df*	*t* value	*p* value
Intercept	**0.938**	**0.290**	**53.01**	**3.235**	**0.002**
Pre-rating	**0.559**	**0.040**	**477.62**	**14.18**	**< 0.001**
*Candidate*					
Le Pen	**0.900**	**0.345**	**494.34**	**2.612**	**0.009**
Other	0.716	0.536	494.98	1.334	0.183
Didn’t vote	0.479	0.298	491.22	1.611	0.108

Random effects	Variance	*SD*
Article (intercept)	0.103	0.321

Model was fit to 500 accuracy ratings from 500 participants across 8 articles. Bolded values indicate significant effects

Replicating Experiments 1 and 2, participants who answered more of the specific details questions correctly on the memory questionnaire gave lower posttest accuracy ratings to the misinformation (*b* = − 1.06, *t*(488.86) = − 2.64, *p* = 0.009). Finally, there was also an effect of article credibility. Participants who rated the article as more credible gave lower accuracy ratings on the posttest (*b* = − 0.25, *t*(496.73) = − 2.14, *p* = 0.033).

### Discussion

The major finding of Experiment 3 was that the fact-checking articles were much less effective for the French participants. Note, however, that *less* effective does not mean *not* effective. Accuracy ratings for the false rumors did decrease after reading the fact check. However, the size of the decrease was much smaller than in the USA and it varied across participants. Participants’ political viewpoint affected their accuracy ratings. Left-leaning participants decreased their belief more than right-leaning participants and Macron voters more so than Le Pen voters. We should note that this does not mean that conservative voters are necessarily more resistant to correction. The current articles were not balanced around whether they would appeal to left or right-leaning participants, as examining who was more or less resistant to correction overall was not a goal of the experiment. We simply take this as evidence that the effectiveness of a fact-checking article will vary based on participants’ prior knowledge and beliefs.

One of the unique visual design features of the CrossCheck fact-checking articles was the use of newsroom logos to indicate that multiple newsrooms agreed with the contents of the article. The goal was to increase the effectiveness of the fact check by increasing its credibility. This experiment suggests that did not work. The fact-checking article was more effective for participants who viewed the article as more credible, but the number of newsroom logos did not affect an article’s credibility or its effectiveness. Participants’ poor memory for the number of logos suggests that they may not have noticed this design feature and may have ignored the logos appearing next to the article. The logos appeared on the right-side of the article in a location where many websites place ads, which may have signaled to readers that the logos were unimportant and could be ignored.

However, it is also possible that the number of newsroom logos did not affect credibility because our news source in the one-logo condition (AFP) was already viewed as credible on its own. The Agence France-Presse (AFP), is a wire service in France that functions like the Associated Press in the USA and is a very large and well-established international news agency. There may have been no need for additional news logos on the articles because AFP was already considered a trusted, non-partisan source. Experiment 4 was designed to rule out this possibility.

## Experiment 4

### Methods

Experiment 4 was very similar to Experiment 3, but it differed in two ways. First, instead of randomly seeing one article from a set of 10 fact-checking articles, all participants viewed the same article. We selected an article about Macron pushing for EU membership for Turkey because it was a “False” article with a medium length (310 words). The major change was in the logo presented in the 1-logo condition. In Experiment 3, we used AFP as the only logo in the 1-logo condition. However, because AFP is a more trusted news source in France, displaying AFP in the 1-logo version could make the article seem just as credible as having four or seven different media logos. Therefore, in Experiment 4 we replaced AFP with a less credible media source, France 24, across all three versions (and replaced France 24 with Les Echos in the 4- and 7-logo versions). The rest of the materials and procedure were identical to Experiment 3.

#### Participants

One hundred and seventy-two adults in France were recruited online using Prime Panels (*M*_age_ = 39 years; age range = 18–75 years). None of the participants had completed Experiment 3. The experiment was conducted in early April of 2018. In the final election, 38% of the participants voted for Macron, 24% voted for Le Pen, 6% voted for a different candidate and 31% did not vote.

### Results

#### Accuracy ratings

Despite the change in newsrooms, the number of logos was again unrelated to participants’ accuracy ratings (Table 12). A mixed-effects regression model predicting accuracy ratings from time (pretest = − 0.5, posttest = 0.5) and logos (1 [reference level], 4, 7) showed a main effect of rating time, (*b* = − 1.04, *t*(169.00) = − 2.65, *p* = 0.008), but no effect of logos, (*b*_4 vs. 1_ = 0.49, *t*(169.00) = 0.87, *p* = 0.387; *b*_7 vs. 1_ = − 0.10, *t*(169.00) = − 0.18, *p* = 0.861), or interaction between logo and time, (*b*_time*4 vs. 1_ = − 0.14, *t*(169.00) = − 0.25, *p* = 0.803; *b*_time*7 vs. 1_ = − 0.21, *t*(169.00) = − 0.38, *p* = 0.708). A simulation-based sensitivity power analysis indicated 80% power, 95% CI [78%, 83%] to detect an interaction between time and the 1 vs 7 logo comparison at least 1.33. The size of the decrease in accuracy ratings (*β* = − 0.31) was similar to the effect size for this particular article in Experiment 3 (*β* = − 0.22) and smaller than relevant decrease for US participants (*β* = − 0.79 Exp 1, *β* = − 1.81 Exp 2).

**Table 12:** Mean accuracy ratings, logo estimates, credibility and bias ratings for the false rumors split by number of logos presented (Experiment 4)

Number of newsroom logos	Pretest accuracy	Immediate posttest accuracy	Estimated logos	Credible	Bias
One	4.88 (3.43)	3.84 (3.23)	2.79 (1.19)	3.02 (1.09)	0.66 (0.48)
Four	5.43 (3.04)	4.26 (3.15)	3.32 (1.40)	2.93 (0.92)	0.53 (0.50)
Seven	4.88 (3.53)	3.64 (3.60)	3.19 (2.24)	2.95 (1.10)	0.60 (0.49)
*M*	5.06 (3.33)	3.91 (3.32)	3.10 (1.68)	2.97 (1.03)	0.60 (0.49)

The accuracy scale ranged from 0 = very inaccurate to 10 = very accurate. Credibility scale ranged from 1 = not at all credible to 5 = extremely credible. Bias is the proportion of participants indicating that the article was biased. Standard deviations are in parenthesis

### Memory questions

For space, we focus here on only participants’ responses to the logo questions, but the full results are presented at https://osf.io/n27pt/. All of the estimates were between 0 and 10 except for one outlier of 88 which was removed. As we only had one observation per participant and one article used in this experiment, we conducted this and all subsequent analyses using linear regression model with fixed effects only. Unlike in Experiment 3, we did not observe even a small significant effect of the number of logos on participants’ logo estimates (*b* = 0.068, *t*(168) = 1.28, *p* = 0.201).

### Credibility and bias

The number of newsroom logos also did not significantly affect participants’ ratings of the credibility of the article, (*b*_4 vs. 1_ = − 0.09, *t*(169) = − 0.45, *p* = 0.655; *b*_7 vs. 1_ = − 0.07, *t*(169) = − 0.36, *p* = 0.721), or if it was biased, (*b*_4 vs. 1_ = − 0.13, *t*(169) = − 1.37, *p* = 0.172; *b*_7 vs. 1_ = − 0.06, *t*(169) = − 0.62, *p* = 0.535).

### Influences on accuracy ratings

Finally, we repeated the analyses from Experiment 3 to see if they replicated with this one rumor about Macron. Participants’ voting choices in the final election did not significantly increase the amount of variance explained when added to a model predicting posttest accuracy ratings given pretest ratings, given in Table 13. However, there was a marginal difference (*p* = 0.052) between Le Pen and Macron voters in their final belief in the rumor (controlling for pretest ratings). Unlike Experiment 3, political orientation did not significantly predict posttest accuracy ratings among people who choose a viewpoint (*b* = 0.02, *t*(126) = 0.176, *p* = 0.861), nor did participants’ ratings of the credibility of the article, (*b* = − 0.005, *t*(169) = − 0.03, *p* = 0.977). But, accuracy on the specific details memory questions was again related to a decrease in posttest accuracy ratings (*b* = − 1.83, *t*(169) = − 2.21, *p* = 0.028).

**Table 13:** Regression analysis testing the effects of candidate choice on posttest accuracy ratings (Experiment 4)

Predictor	Unstandardized *b *(SE)	Standardized *β*	*R*^2^	Δ*R*^2^
Pre-rating	0.60 (0.06)	0.60***	0.38	
Candidate			0.40	0.02
Le Pen	1.02 (0.52)	0.31†		
Other	− 0.33 (0.85)	− 0.10		
Didn’t vote	0.16 (0.48)	0.05		
	Total model *R*^2^ = 0.40, *F*(4, 167) = 27.70, *p* < 0.001			

†*p* < 0.10; ***p < 0.001

### Discussion

We again found that the number of newsroom logos was unrelated to participants’ ratings of the article’s credibility and the effectiveness of the fact-checking article. Similar to Experiment 3, readers had poor memory for the number of logos they saw, indicating that they likely ignored the logos while reading the article. Supporting our conclusion that the effectiveness of a fact check likely depends on an interaction between the reader’s beliefs and the topic of the article, we found differing results in terms of the effect of credibility and political viewpoint in this experiment. Unlike Experiment 3, participants’ political views did not affect their receptiveness toward the article, nor did their ratings of its credibility (despite wide variation in both ratings). While exploring these interactions was not a main goal of this project, future research should examine how participants’ existing beliefs interact with the contents of the article and its effectiveness.

## General discussion

As a reminder, we had three main questions with this research: (1) Does headline style matter for fact-check memory or effectiveness? (2) Do additional newsroom logos increase a fact check’s effectiveness or readers’ trust in the article? and (3) How well do readers remember fact-checking articles? Overall, we found no evidence that the type of headline or number of logos affected readers’ beliefs about the fact-checked claim, their judgements of credibility, or their memory for the article’s content. When measured using multiple-choice questions, participants retained many of the specific details of the article (even 1-week later), but recall was much lower using a free recall measure.

In both US and French samples, reading the fact-checking article decreased belief in the false information. However, the effectiveness varied across the USA and France and across political beliefs within France. There are many possible reasons for the difference in efficacy across the USA and France. One obvious difference is in the participants’ existing knowledge and their investment in the topic. Within the USA, participants were generally unfamiliar with the French election (73% reported either never or only once or twice reading news about the election) and may not have cared whether the presented information was true or false. Within France, participants were both more knowledgeable and more invested in the truth of the rumors. Their preexisting political beliefs and knowledge likely altered how they interpreted the presented information. However, the two countries also likely differ in their baseline trust in news organizations and fact-checking. A recent Pew survey found that 75% of US adults have a lot of or some trust in the information from national news organizations (Pew Research Center, 2019b). A similar survey in France indicated that only 35% of French adults trust the news media a lot or somewhat (Pew Research Center, 2019a).

Within France, we found that the effectiveness of the fact checks sometimes differed based on participants’ political ideology. This particular set of articles was more effective for left-leaning participants and for participants who voted for Macron rather than Le Pen. However, we do not want to overinterpret these differences among the French participants. For one, even when present, the differences were small. In addition, the differences were not always present. Experiment 4, which used only one of the articles did not replicate the effects of political viewpoint found across all of the false rumors in Experiment 3. The current studies were not designed to measure whether left or right-leaning participants were more receptive to fact-checking articles or how participants’ beliefs interact with the topic of the article. The current articles were chosen to be representative of those published by CrossCheck, not to carefully examine the effects of political viewpoint of receptivity to misinformation corrections. Our key takeaway is simply that the articles varied in their effectiveness across different populations.

It is important to note that prior to reading the fact-checking article, the majority of participants were simply uninformed or ambiguous in their beliefs, not misinformed (Li & Wagner, 2020). Across all four experiments, the average pretest accuracy rating for false statements was near or just below the midpoint of the scale indicating that participants were mostly unsure of the truth of the statements initially, with a slight bias to think that they were false. Less than 15% of the US-based participants in Experiments 1 and 2 initially thought that the false headline was very accurate (rating of 8, 9, or 10), while 24% (Exp 3) and 30% (Exp 4) of the participants in France initially believed that the headline was very accurate. Past research has found that it is much easier to update the beliefs of those who are uninformed or unsure (Li & Wagner, 2020). Thus, our findings may not be applicable for populations with more entrenched false beliefs.

Overall, participants had decent memory for the contents of the article (as measured by a multiple-choice test), but they were poor at remembering the visual features and only recalled ~ 15% of the idea units on a free recall test. One’s interpretation of that finding depends on your perspective. On one hand, participants were able to correctly answer 64% of multiple-choice questions about the specific details of the article 1 week later, well above chance. (Note that the exact chance-level performance is unknown. Participants may have been able to achieve higher than chance-level performance with reasonable guesses). On the other hand, even after just reading the article, participants answered 29% of the questions incorrectly. These results suggest that practitioners should make sure to emphasize and possibly repeat the key points that they want the reader to remember. Our readers (who were told to read the article like a typical article on the Internet) failed to remember many of those key points.

We found mixed results about the connection between memory for the contents of the fact-checking article and participants’ accuracy ratings. Across all four studies, there was a significant correlation between the amount of information participants could remember and their rejection of the false rumors. However, when we experimentally manipulated memory accuracy using retrieval practice in Experiment 2, it had no effect on participants’ accuracy ratings 1 week later. Participants who took an immediate memory test were better able to remember the contents of the article 1 week later, but that did not translate into lower accuracy ratings. This finding is still puzzling to us. One reasonable possibility was that increasing memory for the details in the article would help participants remember why the key claim was false, decreasing accuracy ratings. Future research should further explore this connection between memory for the article and belief in its main claim (see Collier et al., 2023 for similar findings and an extended discussion).

Both of the design features tested in these studies (headline style and number of logos) did not affect participants’ perception of the article or the claim. Headlines that were written as a question were similarly effective to headlines written as a negation. Initially, we predicted that the negation headlines may be less effective, especially after 1 week, because participants may have remembered the main claim (e.g., Macron wore and earpiece in the debate), but not the negation. In addition, the question format may have increased readers’ curiosity about whether the rumor was true. However, both types of headlines were similarly effective in reducing belief in the false rumors both immediately and 1 week later and readers remembered both versions similarly.

We also did not see any effect of the number of newsroom logos on article credibility or effectiveness. As demonstrated by poor memory for the number of logos, this is likely because readers did not pay attention to the news articles while reading. Shifting the location of the newsroom logos from the right side of the screen where advertisements are often placed to the top of the article may increase readers’ attention and possibly their perception of credibility. Our results suggest that credibility is important for fact-checking articles (greater credibility ratings were related to lower accuracy ratings for false rumors in Experiment 3), but the newsroom logos did not alter readers’ judgments of credibility. It is important to note that we did not have a no-logo control in any of the experiments. Thus, it is still possible that including at least one newsroom logo increased the effectiveness of the misinformation compared to an article with no logos. However, adding the additional logos did not serve their intended purpose of increasing readers’ perceptions of the article’s credibility and decreasing their perceptions of bias. Of course, there are multiple factors that may affect perceptions of source credibility (e.g., prior knowledge or experience with the source, political affiliations), and thus future research may investigate other factors that affect the credibility of sources of misinformation fact-checks.

We close with some suggestions for both researchers and practitioners. The current studies add to a growing literature that fact-checking articles are generally effective and rarely, if ever, cause backfire effects. Practitioners should feel confident contradicting false information. However, further research is needed on how readers’ existing knowledge, beliefs and trust in media interact with the topic to determine a fact-check’s effectiveness. We observed heterogeneity in the size of the benefit across the US and French samples, but the underlying mechanism driving that difference is currently unclear. We also need further research on the connection between memory for the information contained in a fact-check and belief in the false claim. For practitioners, we believe that more time should be spent thinking about (and empirically testing) what design features readers pay attention to and how that affects their later memory and belief in the false rumor. It is not enough to simply place a label on a fact-check and assume that readers will see it, understand what it means and remember that information. For example, despite placing a label of the type of misinformation on each of the articles, participants correctly recognized only 32% of the labels in Experiment 1. By experimentally testing different article formats, practitioners can learn what is most effective and researchers can better understand how people correct their false beliefs.

## Data Availability

Data, materials and supplemental analyses are available at https://osf.io/n27pt/.

## Footnotes

1 Since there was only one observation per participant, we did not include random intercepts for participants.

2 Note, however, that chance performance may have been higher than 33% if participants were able to accurately guess the answers to some of the questions.
